# Bio-stabilisation of granite residual soil using indigenous microorganisms

**DOI:** 10.1371/journal.pone.0336489

**Published:** 2025-11-10

**Authors:** Ya Wang, Meiqi Li, Hao Peng, Jiaxin Kang, Chenlong Luo, Hong Guo, Yasheng Luo, Mingjiang Tao

**Affiliations:** 1 School of Civil Engineering and Architecture, Shaanxi University of Technology, Hanzhong, China; 2 Student Research Society of Human Settlements, Shaanxi University of Technology, Hanzhong, China; 3 Research Center of Geotechnical Environment and Geological Hazards Control in Qinling-Daba Mountains, Shaanxi University of Technology, Hanzhong, China; 4 College of Biological Science and Engineering, Shaanxi University of Technology, Hanzhong, China; 5 College of Water Resources and Architectural Engineering, Northwest A&F University, Yangling, Shaanxi, China; 6 Department of Civil, Environmental, & Architectural Engineering, Worcester Polytechnic Institute, Worcester, Massachusetts, United States of America; UNICAMP, University of Campinas, BRAZIL

## Abstract

Granite residual soil exhibits inferior mechanical properties, which may lead to slope instability and embankment settlement. Microbial solidification technology offers an environmentally sustainable and highly effective approach for the improvement of such soils. To enhance the strength properties of granite residual soil in the Hanzhong region, three urease-producing Bacillus species, including *Bacillus velezensis*, *Bacillus subtilis*, and *Bacillus tequilensis*, are extracted from the soil in the same area, and solidification improvement experiments are conducted by changing the concentration of the cementing solution. The experimental results indicate that all three bacterial strains can substantially enhance the shear strength of soil. The optimal improvement effect for each strain is observed when the cementing solution concentration reaches 2 mol/L. Notably, *Bacillus subtilis* exhibits the greatest increase in internal friction angle, rising by 145.32% compared to the unimproved. In contrast, *Bacillus tequilensis* shows the highest improvement in cohesion, with a maximum increase of 316.19%. The solidification effect differed among different bacterial strains, with B. *tequilensis* and B. *velezensis* exhibiting better performance in high-concentration cementing solutions. Scanning electron microscopy (SEM) and X-ray diffraction (XRD) analysis reveal that the calcium carbonate precipitates in the soil solidified by these three types of bacteria can strongly bind to the soil particles, confirming the improvement effect at the microscopic level. This study provides an eco-friendly and cost-effective improvement method for the engineering application of granite residual soil, which plays an important role in improving the quality and decreasing the cost of artificial slope filling, roadbed filling, and foundation pit backfilling in areas with granite residual soil.

## 1. Introduction

Granite residual soil is widely distributed in southeastern and southwestern China and is characterized by a low clay content, a high degree of weathering, and strong water sensitivity. Under rainy weather, **granite residual soil** is prone to disintegration, which can significantly decrease strength and cause disasters such as instability of roadbed slopes, thus posing a serious threat to the safety of engineering construction. Therefore, as an important roadbed filling material, granite residual soil needs to be improved to meet engineering requirements. Commonly used soil improvement methods include physical compaction, reinforcement improvement, and admixture improvement [1–7]. However, physical compaction and reinforcement improvements require a large amount of energy and artificial materials, resulting in high engineering costs. The use of some materials as external additives may have unsustainable negative effects on the ecological environment [8]. Microbial-induced calcium carbonate precipitation (MICP) technology has rapidly developed and achieved significant results in the field of geotechnical engineering because of its advantages of small disturbances, energy conservation, and environmental protection [9]. This technology uses the metabolic process of microorganisms to generate calcium carbonate precipitation through biochemical reactions. These **precipitates** adhere to the pores of the soil, fill the pores, and bond soil particles, thereby improving the strength and stability of the soil. Therefore, MICP technology provides a more sustainable and eco-friendly solution for improving soil.

Many researchers have applied MICP technology to improve various types of soil, such as Tiwari et al. [10], who treated expansive soil with MICP and reported that the compressive strength of soil samples treated with MICP increased by more than 205% compared to that of untreated samples. Shi et al. [11] used MICP technology to reinforce CQU-L1 and CQU-L2 simulated lunar soil and reported that increasing the number of reinforcements and the concentration of cementing solution can significantly improve the strength of lunar soil and improve the pore structure. Zamani and Montoya [12] reported that MICP technology effectively improved the undrained shear response of fine-grained sandy soil, decreased the excess pore water pressure, and improved the shear strength. Liang et al. [13] improved the strength of **granite residual soil** using MICP technology and reported that MICP treatment can significantly increase the shear strength, internal friction angle, and cohesion of the soil.

In the improvement experiment, the uniformity of the bacterial liquid distribution in the soil was also an important factor affecting the strength of the soil. Dai et al. [14] conducted MICP tests on standard sand using *Sporosarcina pasteurii* and reported that direct injection and alternating injection methods can achieve a uniform distribution of bacterial solutions, but the injection of cementing solution can wash away bacteria and affect the uniformity of reinforcement. Tobler et al. [15] studied the effects of different injection methods on sand reinforcement and reported that cyclic injection of bacterial solution and cementing solution can further improve uniformity and sample strength.

Some researchers also added additives and reinforcement materials to the soil to enhance the improvement effect through the joint action of bacteria. Liu et al. [16] designed and conducted experiments on carbonic anhydrase-enhanced microbial mineralization solidification of sandy soil. They significantly increased the yield of cement during the microbial solidification process of sandy soil, thereby increasing the compressive strength and deformation resistance of the solidified body. Zhang et al. [17] proposed an unsaturated reinforcement method based on microbial induced calcite precipitation (MICP) combined with short fibers (1–5 mm). Through the preparation of composite sand columns, it was found that short fibers significantly improved the unconfined compressive strength and ductility of sand columns, and the effect was better under unsaturated conditions.

Furthermore, the concentration of the cementing solution and the type of calcium source are critical factors that significantly influence the properties of the stabilized soil. Huang et al. [18] conducted MICP technology treatment experiments on sandy loess using three sources of calcium: calcium chloride, calcium acetate, and calcium lactate. They reported that calcium acetate had better solidification uniformity as a calcium source, whereas calcium lactate had higher early strength, but calcium chloride had better later strength. Zhang et al. [19] improved granite residual soil by changing the concentration of the cementing solution. By conducting unconfined compressive strength tests, calcium carbonate generation tests, and SEM microstructure analysis, it was found that the cementing solution at a concentration of 1.0 mol/L had the best reinforcement effect, and the soil column sample had the highest strength.

Based on the above analysis, researchers achieved significant results in MICP improvement of various types of soils from different perspectives by purchasing freeze-dried powder or bacterial solutions. In this study, granite residual soil from the Hanzhong area was examined, focusing on the feasibility of extracting local bacterial strains for improvement. Three MICP strains capable of producing urease, namely, *Bacillus subtilis*, *Bacillus tequilensis*, and *Bacillus velezensis*, were extracted from local soil. Considering the effect of cementing solution concentration, the improvement effect of cementing solution on granite residual soil was investigated through direct shear tests. Finally, the solidification mechanism was explained through X-ray diffraction (XRD) and scanning electron microscopy (SEM) experiments. The results obtained provided new ideas for improving granite residual soil roadbed slopes, which have practical significance and promising application prospects.

## 2. Experimental materials

### 2.1. Ethical statement

The field sampling for this study was carried out on publicly accessible land that is neither privately owned nor subject to environmental or cultural protection regulations. No specific permits were required for access or sample collection at these sites, as verified through a review of relevant local regulations. The study did not involve any endangered or protected species. All necessary measures were taken to minimize disturbance to the sampling environment.

### 2.2. Physical properties of granite residual soil

**Fig 1:**
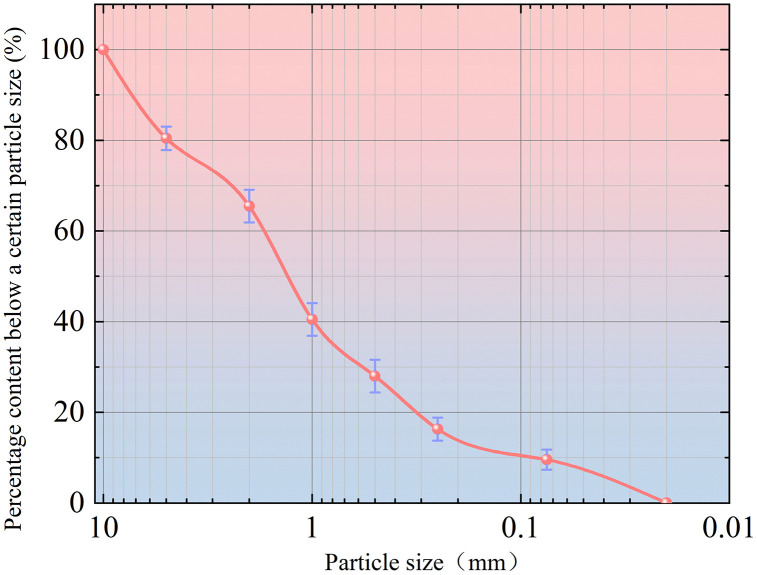
Particle size distribution curve of granite residual soil.

The granite residual soil used in this study was collected from a highway slope in Chenggu County, Hanzhong City, Shaanxi Province, and the collected soil samples were dried. Following the “Standard for Soil Test Methods” (GBT50123−2019) [20], standard compaction tests and limit moisture content tests were conducted on the soil samples. The maximum dry density of granite residual soil was 2 g/cm^3^, the optimal moisture content was 9.8%, the liquid limit was 16.8%, and the plastic limit was 10.5%. To ensure the reliability of the test results, three sets of parallel particle analysis tests were conducted, and the particle size gradation curves for the soil samples were obtained through **sieve analysis**. The results are illustrated in Fig 1. The data points presented in the figure represent the average values derived from three repeated experiments.

### 2.3. Culture medium and cementing solution

(1) **Culture medium**

When screening and purifying bacterial strains, specific culture media are needed. The screening and isolation media used in this study consisted of peptone (1 g/L), sodium chloride (5 g/L), potassium dihydrogen phosphate (2 g/L), glucose (1 g/L), urea (20 g/L), phenol red (0.012 g/L), and agar (15 g/L), with a pH of 7.0 ± 0.5. Urea and glucose were separately prepared into solutions, filtered and sterilized, and added to sterilized culture media. The Luria-Bertani medium (pH 7.0 ± 0.5) used for preserving the purified bacterial strains consisted of 10 g/L tryptone, 5 g/L yeast extract, 10 g/L sodium chloride, and 15 g/L agar.

(2) **Cementing solution**

We used a mixed solution of calcium chloride and urea as the cementing solution; calcium chloride served as a source of calcium for the solidification reaction, and urea was hydrolyzed by urease to generate carbonate ions. The cementing solution was prepared by mixing calcium chloride, urea, and distilled water in the following proportions: 0.5 mol/L cementing solution (1000 mL distilled water, 55.5 g calcium chloride, and 30.03 g urea), 1 mol/L cementing solution (1000 mL distilled water, 111 g calcium chloride, and 60.06 g urea), and 2 mol/L cementing solution (1000 mL distilled water, 222 g calcium chloride, and 120.12 g urea) [21].

## 3. Experimental methods and processes

### 3.1. Screening and purification of urease-producing strains

The soil sample was passed through a 60 mesh sieve, 25 g was weighed, and the sample was placed in a conical flask containing 225 mL of sterile water. The mixture was allowed to stand for 15 min in an 80°C water bath. The supernatant was then diluted to 10⁻⁶ . Subsequently, 100 μL of each dilution solution from the tubes (10^-4^, 10^-5^, and 10^-6^) was inoculated into the screening and isolation media in ten replicate samples. The inoculated media were then incubated at 30°C for 24–48 hours. The screening and isolation media contained urea and phenol red as indicators. The urease-producing strain hydrolyzes urea to produce alkaline ammonia, and under the action of the phenol red indicator, the colony appears purple-red around it. Color-changing bacterial strains were selected and purified by streaking on screening and isolation media, with each strain subjected to three replicate purifications. The cultures were incubated at 30°C for 24 hours, after which individual colonies were picked and inoculated into Luria-Bertani (LB) slant medium for long-term preservation. After screening, three strains were identified by molecular biology, namely, B. *subtilis*, B. *tequilensis*, and B. *velezensis*.

**Fig 2:**
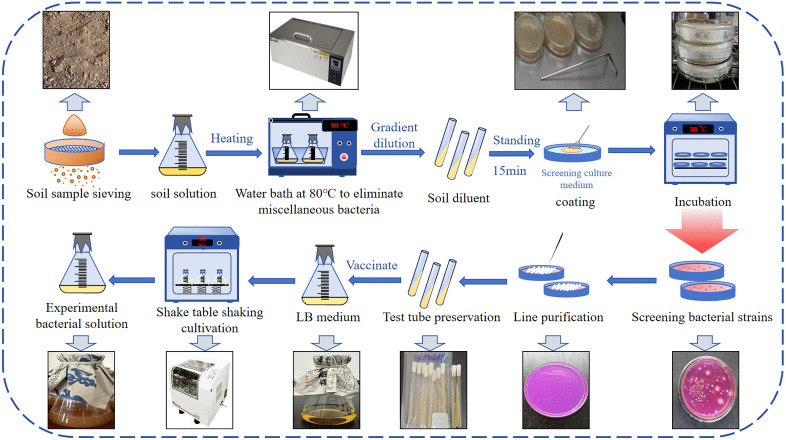
Bacterial screening and bacterial solution cultivation.

Before the experiment, the three types of bacteria in the slant medium were inoculated in Luria-Bertani liquid medium and cultured at 30°C for 24–48 hours. The activation culture was performed in triplicate for each strain, and turbidity of the solution was observed. If turbidity was found, the strain was considered to be activated. The specific process of strain screening and bacterial culture is shown in Fig 2. The average absorbance was measured using a UV-visible spectrophotometer at 600 nm, and the OD_600_ value was 1.731, which indicated that the bacterial mixture had reached its optimal growth state and could be used for MICP improvement experiments.

### 3.2. Microbial improvement of the granite residual soil test

**Fig 3:**
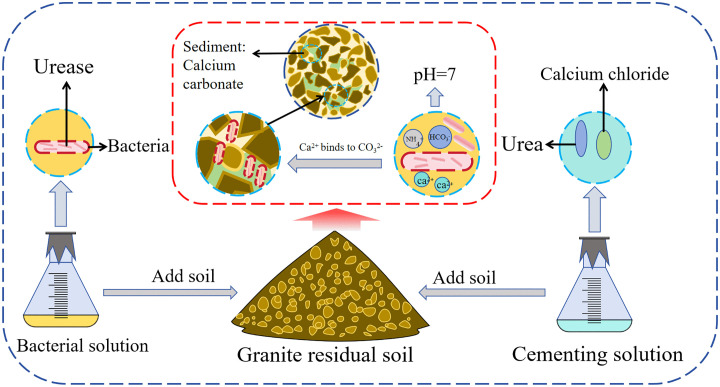
Illustration of the improved solidification process.

The improvement experiment was conducted using the mixing method [21]. After completely drying the retrieved granite residual soil, it was sieved, and soil samples with a particle size below 2 mm were taken based on the particle size distribution for future use. The soil sample, cementing solution, and bacterial solution were prepared in a mass-to-volume ratio of 500 g: 100 mL: 50 mL. To ensure homogeneous distribution of the bacterial and cementing solutions within the soil matrix, a two-stage mixing procedure was implemented. Initially, the bacterial solution was introduced into the soil sample and mixed for 5 minutes until uniformly dispersed. Subsequently, the cementing solution was added and blended for an additional 5 minutes. Following complete homogenization, the mixture was transferred into a temperature-controlled chamber maintained at 30°C and cured for 14 days. The improvement experiment used B. *tequilensis*, B. *subtilis*, and B. *velezensis* as solidification strains and was conducted with cementing solution concentrations of 0, 0.5, 1.0, and 2.0 mol/L, respectively. The specific experimental plan and soil sample numbers are presented in Table 1. Among these, three parallel samples were established for each experimental group to ensure the reliability and repeatability of the data. After solidification, the soil sample was loaded into the mold to prepare the sample. The specific solidification process and mechanism are shown in Fig 3.

**Table 1:** Experimental scheme.

Bacterial strain	Concentration of cementing solution/(mol/L)	Number
--	--	Granite residual soil
*Bacillus velezensis*	0.5	J1N1
	1	J1N2
	2	J1N3
*Bacillus subtilis*	0.5	J2N1
	1	J2N2
	2	J2N3
*Bacillus tequilensis*	0.5	J3N1
	1	J3N2
	2	J3N3

### 3.3. Direct shear test

The direct shear test was conducted using the **ZJ strain-controlled direct shear apparatus** manufactured by Nanjing Soil Instrument Factory. The sample had a height of 20.00 mm, a diameter of 61.8 mm, and a dry density of 1.8 g/cm^3^. The vertical pressures applied in the test were 100 kPa, 200 kPa and 300 kPa respectively. Three parallel specimens were prepared under each pressure condition, with a shear rate of 0.8 mm/min.

### 3.4. SEM and XRD tests

The SEM experiment was conducted using a German ZEISS Sigma 300 scanning electron microscope. The XRD experiment was conducted using an X-ray diffractometer (Rigaku SmartLab SE) with diffraction angles between 5° and 90°.

## 4. Results and discussion

### 4.1. Shear stress-shear displacement curve

(1) **Analysis of the effect of different bacterial strains**

**Fig 4:**
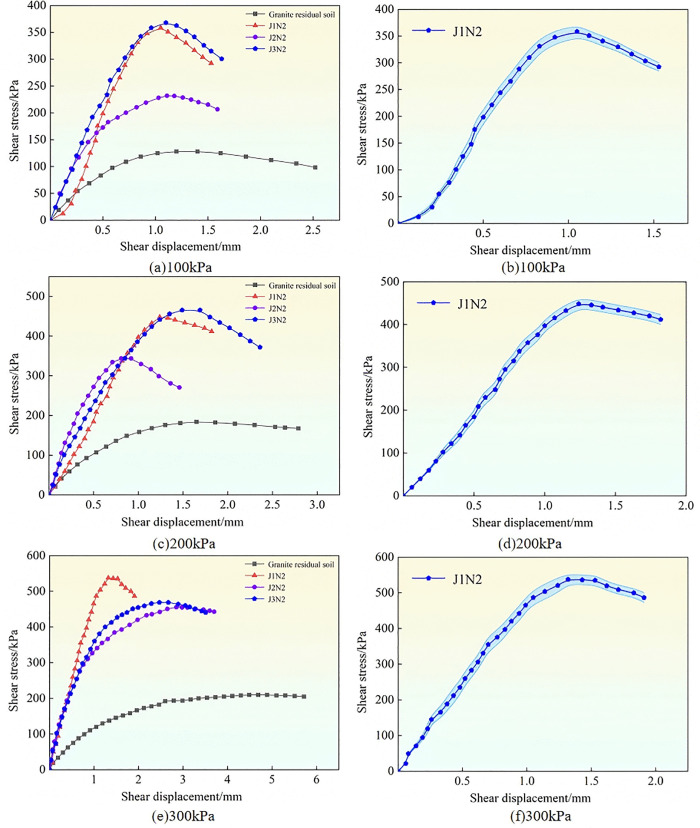
Shear stress-shear displacement curves of modified samples with different bacterial strains.

Using a cementing solution with a concentration of 1 mol/L as an example, the shear stress-shear displacement behavior of granite residual soil treated with three types of bacteria across three parallel tests is shown in Fig 4. To verify the reliability of the results, sample J1N2 was selected as a representative case. **The shear stress-shear displacement relationships from the three replicate tests, along with their associated error ranges, are presented in** Figs 4(b), (d), and (f).

According to Fig 4, when the vertical stress was 100 kPa, 200 kPa, or 300 kPa, the shear stress-strain curves of the three bacterial treatments were greater than those of the untreated soil, which indicated that the soil modified by bacteria had greater shear stress. As the shear displacement increased, the shear stress first increased and then decreased, exhibiting strain-softening characteristics. Under vertical stress of 100 kPa and 200 kPa (Figs 4 (a) and (c)), the shear stress of B. *velezensis* and B. *tequilensis* (J1 and J3) increased significantly and similarly, whereas the increase was slightly smaller for B. *subtilis* (J2). When the vertical stress was 300 kPa (Fig 4 (e)), the peak shear stress of granite residual soil treated with B. *velezensis* (J1) was greater than that of the other two strains.

(2) **Analysis of different cementing solution concentrations**

**Fig 5:**
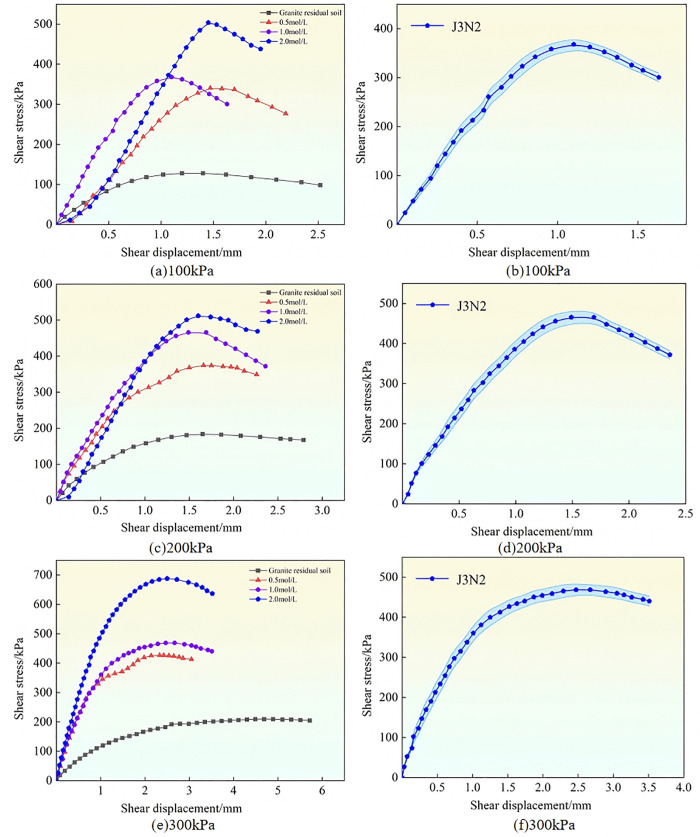
Shear stress-shear displacement curves of modified samples with different concentrations of cementing solution.

Taking B. *tequilensis* as an example, the shear stress-shear displacement behavior of the improved granite residual soil under different concentrations of cementing solution across three parallel tests is illustrated in Fig 5. To verify the reliability of the results, sample J3N2 was selected as a representative case. The shear stress-shear displacement relationship **from the three replicate tests, along with their associated error ranges, are presented in** Figs 5(b), (d), and (f),.

As shown in Fig 5, when the vertical stress was 100 kPa, 200 kPa, or 300 kPa, the soil sample exhibited strain-softening characteristics as the concentration of the cementing solution increased. Among them, the shear stress of the soil was the highest when the concentration of the cementing solution was 2.0 mol/L. This occurred because the higher the concentration of the cementing solution, the greater the content of calcium carbonate precipitate generated by reacting with the bacterial mixture, and the greater the shear stress of the soil. Under 300 kPa vertical stress (Fig 5 (e)), the peak shear stress of the sample with a concentration of 2.0 mol/L cementing solution increased continuously, whereas the peak shear stress of the sample with a concentration of 0.5 mol/L cementing solution increased slightly, and the peak shear stress of the sample with a concentration of 1.0 mol/L cementing solution hardly increased.

### 4.2. Shear strength index

**Fig 6:**
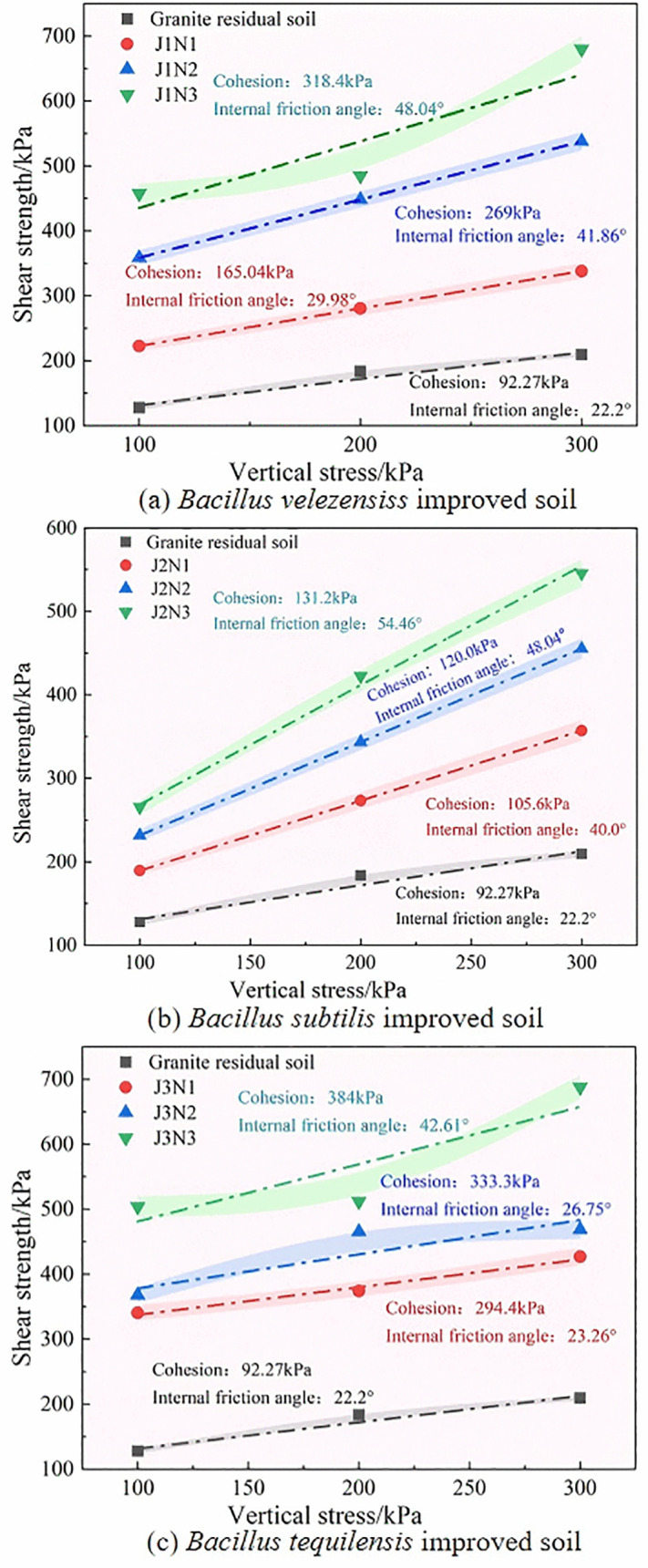
Shear strength-vertical stress curves of different bacterial strains.

**Fig 7:**
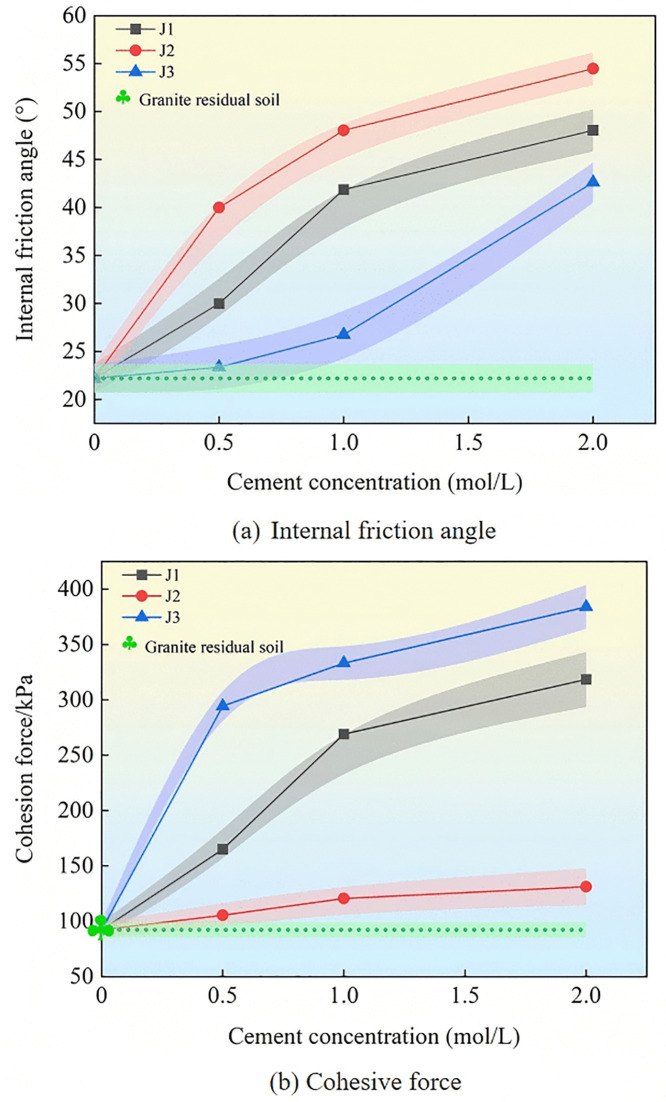
Strength parameters of improved granite residual soil.

The relationship curve between the shear strength and vertical stress in the direct shear test is shown in Fig 6. The calculated shear strength index after linear fitting is illustrated in Fig 7.

The cohesion and internal friction angle of granite residual soil samples improved by different bacterial strains were significantly greater than those of untreated soil (Fig 6). These results confirmed that all three improved bacterial strains can effectively improve the shear resistance of granite residual soil. The different bacterial strains significantly affected the shear strength of soil samples, with soil samples treated with B. *tequilensis* or B. *velezensis* exhibiting greater shear strength than those treated with B. subtilis. The soil sample with a cement concentration of 2 mol/L had the highest shear strength.

As shown in Fig 7, B. *velezensis* and B. *subtilis* (J1 and J2) can significantly increase the internal friction angle of soil compared to B. *tequilensis* (J3), whereas B. *velezensis* and B. *tequilensis* (J1 and J3) can significantly improve the cohesion of soil compared to B. *subtilis* (J2). As shown in Fig 7 (a), as the concentration of the cementing solution increased, the internal friction angle of the granite residual soil improved because the three strains of bacteria increased. Among them, B. *velezensis* and B. *subtilis* (J1 and J2) showed greater increases at cementing solution concentrations of 0.5 mol/L and 1.0 mol/L. When the concentration of the cementing solution was 2 mol/L, the internal friction angle of B. subtilis (J2) was the highest, with an increase of 145.32% compared to that of the untreated soil. As shown in Fig 7 (b), the cohesion of B. *velezensis*, B. *subtilis*, and B. *tequilensis* (J1, J2, and J3) increased with an increase in the concentration of the cementing solution. Under different concentrations of cementing solution, B. *tequilensis* (J3) presented the best cohesion among the three bacteria. When the concentration of the cementing solution was 2 mol/L, the cohesion of B. *tequilensis* increased by a maximum of 316.19% compared to that in the untreated soil.

Overall, when the concentration of the cementing solution was 2 mol/L, the internal friction angle and cohesion of the soil samples improved as the three bacterial species reached their maximum values. This occurred because the calcium carbonate content generated by the cementing solution at low concentrations was limited, resulting in a weak bonding force between the soil particles in the soil. As the concentration of the cementing solution increased, the content of calcium carbonate generated increased, the number of soil particle pores decreased, and the internal friction angle and cohesion increased.

### 4.3. XRD pattern analysis

**Fig 8:**
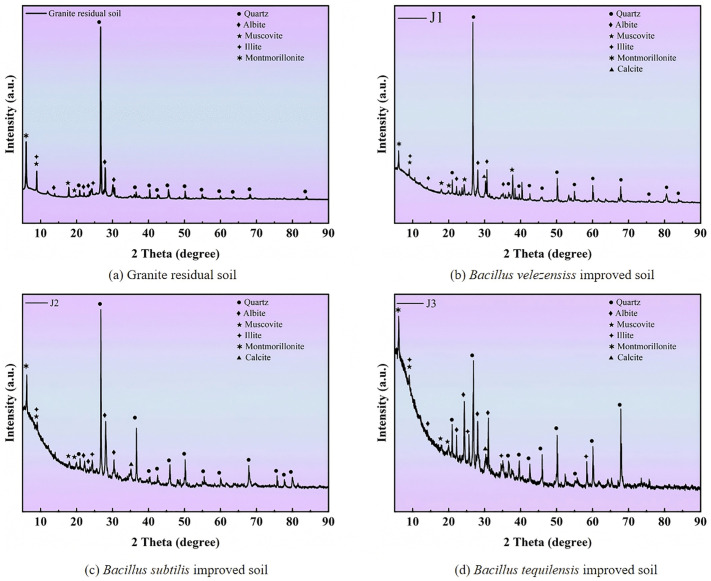
XRD pattern of improved granite residual soil.

Based on the results of the direct shear test, samples of improved granite residual soil prepared with a cementing solution concentration of 2 mol/L were selected for X-ray diffraction analysis to determine the mineral composition before and after improvement, as shown in Fig 8. All characteristic peaks in the spectrum were identified by comparison with the JCPDS standard cards. The corresponding reference codes for each mineral phase are as follows: Albite: 41–1480, Calcite: 05–0586, Muscovite: 34–0175, Illite: 29–1496, Quartz: 87–2096.

A comparison of the mineral PDF cards revealed that the main mineral components of the untreated soil are quartz, albite, muscovite, illite, and montmorillonite (Fig 8 (a)). As shown in Figs 8 (b), (c), and (d), the mineral composition of the soil sample improved by the three types of bacteria underwent significant changes, with the newly added mineral being calcite. These findings indicated that the three bacterial species induced significant precipitation of calcium carbonate.

### 4.4. SEM microstructure analysis

**Fig 9:**
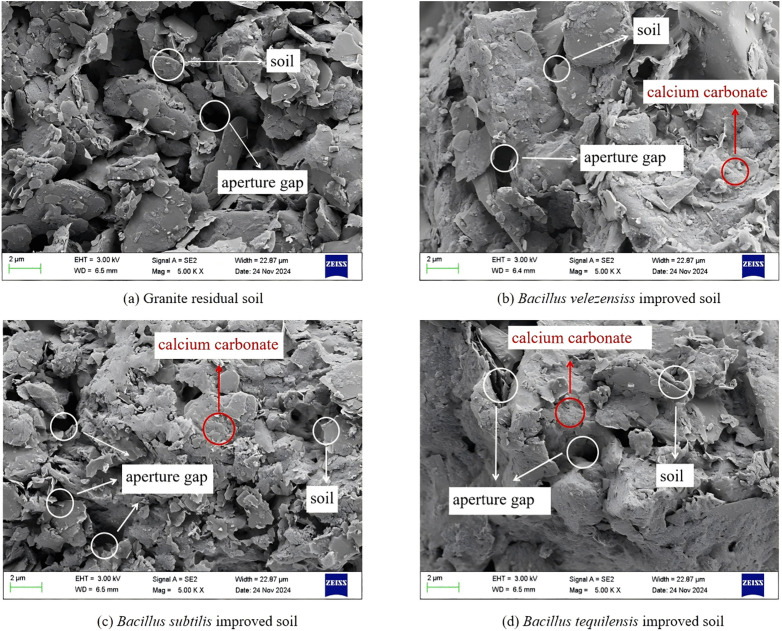
SEM images of different strains before and after cementation.

The microstructure characteristics of **granite residual soil** before and after improvement were identified by SEM experiments (Fig 9).

The microstructure of the unmodified soil particles was mainly in the form of sheet-like structures, with relatively dispersed particles and no prominent aggregate structure. The pore space was relatively large (Fig 9 (a)); there were many aggregates distributed widely among the soil particles improved by B. *velezensis* and B. *tequilensis*, with significantly reduced soil pores, calcium carbonate deposition, and enhanced particle bonding (Figs 9 (b) and (d)). There were some aggregates between soil particles improved by B. *subtilis*, but their size was small, and their distribution was limited, resulting in some deposition of calcium carbonate (Fig 9 (c)). The MICP improvement of the three types of bacteria resulted in the formation of calcium carbonate “cement bodies”, which increased the compactness of the soil sample and the bonding force between particles, thereby significantly improving its shear strength.

## 5. Discussion

Although this study demonstrated the efficacy of indigenous microorganism-induced calcium carbonate precipitation (MICP) technology in enhancing the shear strength of granite residual soil in the Hanzhong region, several limitations remain to be addressed:

(1) While SEM analysis has partially elucidated the distribution of cementing materials, a quantitative evaluation of the spatial uniformity of biological cementing agents within the soil matrix remains lacking, which warrants further investigation in future studies.
(2) The experimental design primarily focused on the potential of soil improvement using indigenous microorganisms, but did not include assessments of critical engineering properties such as durability, unconfined compressive strength, permeability, and leaching behavior. Future work will emphasize the systematic evaluation of these key performance indicators.
(3) This study was specifically conducted on granite-derived residual soils in the Hanzhong region, and soil properties in other geographical areas may differ significantly. Furthermore, parameters such as temperature, bacterial solution concentration, and the pH value of the cementing solution were not evaluated as potential influencing factors in the current work. Future research will aim to investigate the underlying mechanisms of these variables and extend the geographical applicability of the findings.

## 6. Conclusions

In this study, we verified the feasibility of using three self-extracted bacterial species (B. *velezensis*, B. *subtilis*, and B. *tequilensis*) to solidify and improve granite residual soil in the Hanzhong area through MICP technology, providing a new technique for reinforcing and improving granite residual soil. The main conclusions of the study are as follows:

(1) *Bacillus* *velezensis*, B. *subtilis*, and B. *tequilensis* significantly improved the shear strength of granite residual soil in the Hanzhong area, with B. *tequilensis* showing the best performance. As the concentration of the cementing solution increased, the improvement effect on the three bacteria increased. When the concentration of the cementing solution was 2 mol/L, its shear strength reached its peak value.
(2) An increase in the internal friction angle and cohesion of the soil effectively increased the shear strength of granite residual soil. *Bacillus* *velezensis* and B. *subtilis* had significant effects on increasing the internal friction angle, whereas B. *velezensis* and B. *tequilensis* had greater effects on increasing cohesion.
(3) Scanning electron microscopy (SEM) and XRD experiments revealed that large amounts of calcium carbonate cement formed in the granite residual soil after MICP improved, and the pore size decreased. The three bacterial species significantly increased the bonding force and compactness between particles in the soil, leading to an increase in the internal friction angle and cohesion, which in turn increased its shear strength.

Future research should prioritize a comprehensive evaluation of the economic viability, environmental implications, and scalability potential of indigenous microorganisms in practical engineering applications, in order to systematically advance their real-world application value.

## Supporting information

S1 File Inclusivity-in-global-research-questionnaire. (DOCX)

S1 Data Minimum dataset. (ZIP)
